# Application of Real-Time Palm Imaging with Nelder–Mead Particle Swarm Optimization/Regression Algorithms for Non-Contact Blood Pressure Detection

**DOI:** 10.3390/biomimetics9110713

**Published:** 2024-11-20

**Authors:** Te-Jen Su, Ya-Chung Hung, Wei-Hong Lin, Wen-Rong Yang, Qian-Yi Zhuang, Yan-Xiang Fei, Shih-Ming Wang

**Affiliations:** 1 Department of Electronic Engineering, National Kaohsiung University of Sciences and Technology, Kaohsiung 807618, Taiwan; sutj@nkust.edu.tw (T.-J.S.); i110152101@nkust.edu.tw (Y.-C.H.); i110152102@nkust.edu.tw (W.-H.L.); i110152105@nkust.edu.tw (W.-R.Y.); j111252102@nkust.edu.tw (Q.-Y.Z.); f111152138@nkust.edu.tw (Y.-X.F.); 2 Department of Computer Science and Information Engineering, Cheng Shiu University, Kaohsiung 833301, Taiwan

**Keywords:** blood pressure, non-contact monitoring, independent component analysis, Nelder–Mead simplex method, particle swarm optimization algorithm, machine learning, regression analysis

## Abstract

In response to the rising prevalence of hypertension due to lifestyle changes, this study introduces a novel approach for non-contact blood pressure (BP) monitoring. Recognizing the “silent killer” nature of hypertension, this research focuses on developing accessible, non-invasive BP measurement methods. This study compares two distinct non-contact BP measurement approaches: one combining the Nelder–Mead simplex method with particle swarm optimization (NM-PSO) and the other using machine learning regression analysis. In the NM-PSO method, a standard webcam captures continuous images of the palm, extracting physiological data through light wave reflection and employing independent component analysis (ICA) to remove noise artifacts. The NM-PSO achieves a verified root mean square error (RMSE) of 2.71 mmHg for systolic blood pressure (SBP) and 3.42 mmHg for diastolic blood pressure (DBP). Alternatively, the regression method derives BP values through machine learning-based regression formulas, resulting in an RMSE of 2.88 mmHg for SBP and 2.60 mmHg for DBP. Both methods enable fast, accurate, and convenient BP measurement within 10 s, suitable for home use. This study demonstrates a cost-effective solution for non-contact BP monitoring and highlights each method’s advantages. The NM-PSO approach emphasizes optimization in noise handling, while the regression method leverages formulaic efficiency in BP estimation. These results offer a biomimetic approach that could replace traditional contact-based BP measurement devices, contributing to enhanced accessibility in hypertension management.

## 1. Introduction

Hypertension has always been one of the most critical potential factors causing death and increasing medical burden around the world. The World Hypertension Federation calls on governments to pay attention to measuring blood pressure. Because high blood pressure is a common risk factor for major chronic diseases such as cardiovascular disease, stroke, diabetes, and kidney disease, blood pressure (BP) can reflect the underlying physical condition. It refers to the side diameter of the blood in the blood vessel per unit area. Pressure and blood pressure are affected by many factors, such as height, weight, age, mood, blood density, etc., so blood pressure values constantly change within a specific range. If the blood pressure is too low, blood may not be supplied to the whole body smoothly; if the blood pressure is too high, the blood vessels may be injured. Testing the blood pressure value can reflect whether the heart function is abnormal. Hypertension has always been a health issue of great concern to people, and it is also one of the diseases that medical and health units in various countries have actively invested resources in prevention and treatment for a long time. Hypertension is generally asymptomatic, so it is known as the “silent killer.” A few patients may have symptoms such as headache, difficulty breathing, or nosebleeds, which are usually quite severe. Therefore, the only way to know whether you have high blood pressure is to measure your blood pressure regularly every day.

In related references, various approaches have been explored to address non-contact blood pressure (BP) measurement with different levels of accuracy and limitations. Amal E. et al. [1] introduced a multi-stage deep neural network model utilizing photoplethysmography (PPG) signals for BP estimation. Despite promising results, PPG-based methods often rely on close contact or optimal lighting conditions, which limit their applicability in real-world, non-contact scenarios. Nicolas A. et al. [2] proposed a cuffless arterial BP monitoring approach based on a seq2seq deep learning model and attention mechanism using finger PPG signals. While their approach yielded reasonable accuracy (MAE: 6.57 ± 0.20 mmHg for DBP, 14.39 ± 0.42 mmHg for SBP) on a large-scale dataset, demographic diversity and the absence of direct contact present challenges for consistent performance across different populations. Similarly, Aguirre N. et al. [3] developed a method based on iPPG signals from camera-based forehead images. However, iPPG’s dependence on lighting and facial visibility can impact measurement accuracy, as indicated by their DBP MAE of 8.36 ± 6.22 mmHg and SBP MAE of 5.69 ± 3.97 mmHg. Other studies, such as Stogiannopoulos T. et al. [4], have explored infrared imaging combined with motion magnification, which demonstrated accuracy in low-light conditions but remains limited in broad applicability due to specific hardware requirements. Further non-contact BP models, such as Fang H. et al. [5], combined iPPG with CNN, BiLSTM, and GRU to process facial images. Despite achieving respectable accuracy (MAE: 12.35 mmHg for SBP, 9.54 mmHg for DBP) across various demographics, the need for consistent light sources and facial stability affects robustness. Cheng H. et al. [6] and Stephanie B. et al. [7] introduced multi-stage models that integrate CNN and recurrent networks for BP estimation using iPPG and ECG signals, though these methods still face environmental and demographic challenges. Goudarz R. H. et al. [8] used a full-wave frequency method on green and red channel iPPG signals, achieving low error rates but noting limitations in signal consistency across skin tones and lighting conditions. Collectively, these studies highlight the potential and limitations of current non-contact BP measurement methods, particularly in handling lighting variability, noise, and demographic diversity. This study addresses these limitations by proposing two robust methods—NM-PSO and regression—designed to improve accuracy and applicability across varied conditions, offering a practical advancement in non-contact BP monitoring. This work investigated two advanced non-contact blood pressure measurement methods. First, the area of the palm is captured through the webcam, and the sample signal of the green wavelength in the light wave reflection is taken out. The first method uses a hybrid Nelder–Mead and particle swarm optimization algorithm to create a different BMI. The empirical parameters of the blood pressure formula in the interval are finally calculated; the second method uses machine learning and regression analysis to calculate the formula statistically and then inserts it into physiological values to calculate the blood pressure value. The above method builds a non-contact blood pressure measurement system, which is expected to be applied to mobile devices in the future, saving the cost of purchasing blood pressure machines and becoming an auxiliary tool that can monitor one’s blood pressure anytime and anywhere without having to carry a bulky blood pressure machine. According to the standards of the British Hypertension Society (BHS) and the Association for the Advancement of Medical Instrumentation (AAMI), our model’s performance meets the AAMI standards requirements. In addition, according to the BHS standards, the model achieved grade A in estimating systolic and diastolic blood pressure. The mean and standard deviation errors in systolic and diastolic blood pressure estimates were +1.91 ± 5.55 mmHg and +0.67 ± 2.84 mmHg, respectively.

The structure of this paper is as follows: Section 2 details the related theories, Section 3 details the research methods and systems description, and Section 4 shows the experimental results. The experimental results are discussed in Section 5 and Section 6, and this study’s main findings and contributions are summarized, respectively.

## 2. Related Theories

The NM-PSO and regression methods proposed in this study are both inspired by biological behaviors and principles of natural selection. First, the particle swarm optimization (PSO) component mimics the collective behavior observed in bird flocks and fish schools, where individuals (particles) adjust their positions based on their own experiences and the successes of their peers. This mechanism reflects how organisms in natural ecosystems cooperate and adapt to optimize resource utilization. Additionally, the Nelder–Mead simplex method incorporates a deterministic approach similar to the refinement processes found in evolutionary systems, where gradual improvements occur through mechanisms akin to “survival of the fittest”. By combining these two methods, NM-PSO leverages the exploratory capabilities of PSO to avoid local optima while using the Nelder–Mead method for precise solution adjustments, achieving a balance between exploration and exploitation.

Meanwhile, the regression method emulates the ability of biological systems to learn patterns and relationships in data. Built on the foundation of multivariate analysis, this method quantifies linear relationships between input and output variables, enabling rapid and accurate blood pressure estimation. This approach is analogous to how biological organisms statistically analyze environmental cues to make quick decisions.

By integrating NM-PSO and regression methods, our system achieves robust handling of dynamic data and enhanced computational efficiency, demonstrating the powerful practicality of biomimetic principles. Each method has distinct strengths: NM-PSO excels in noise resilience, while the regression method offers faster computations, making them suitable for various application scenarios.

### 2.1. Hand Tracking Technology, Region of Interest (ROI), and Normalization

**Figure 1:**
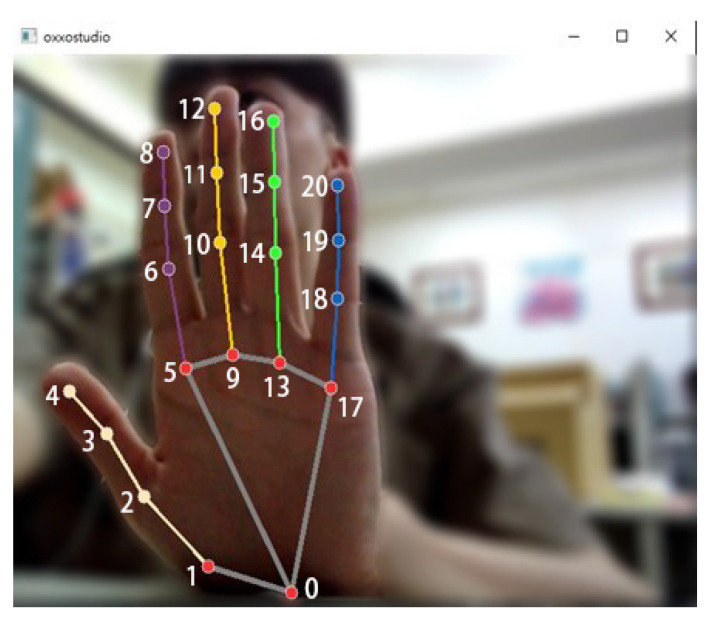
Schematic diagram of MediaPipe hand node model.

This work uses MediaPipe, a cross-platform machine learning framework developed by Google, to recognize and track the hand. MediaPipe specializes in visual processing applications and is based on CNN technology, utilizing deep learning for high-precision analysis and tracking of hand images. When detecting the hand, MediaPipe generates 21 landmarks on the palm and fingers, each corresponding to a key knuckle or joint, as illustrated in Figure 1. These landmarks provide a detailed map of the hand’s structure, allowing precise tracking of hand gestures and positioning, which is crucial for consistent measurement accuracy in this study. The specific names of each landmark point are provided in Table 1 [9,10,11].

**Figure 2:**
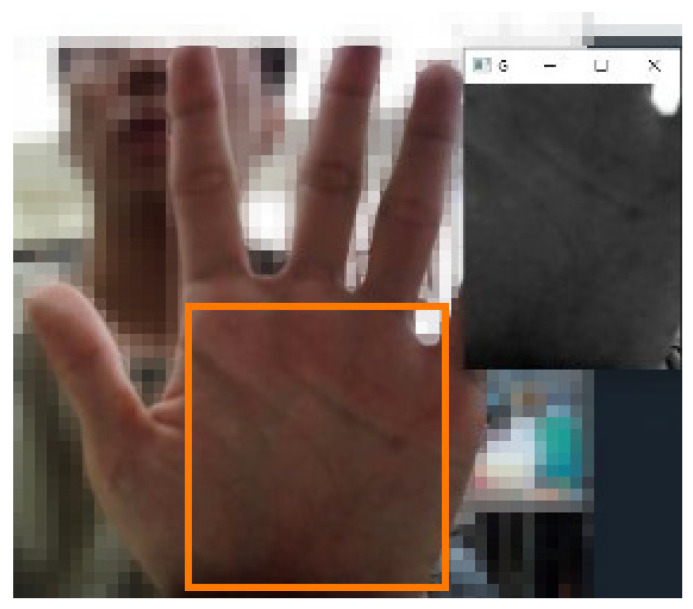
Diagram of the area of interest.

To accurately capture the region of interest (ROI) for blood pressure measurement, we define the palm area as the primary target for data collection, as shown in Figure 2. The palm area is selected because it provides a stable and consistent blood pulse signal, less susceptible to interference from non-skin regions or finger movements. Specifically, four key landmarks (points 1, 5, 17, and 0) are used to outline the ROI to ensure the most relevant area of the hand is captured.

Since raw data may vary in units and ranges, direct analysis could lead to inconsistencies. Therefore, normalization is applied to standardize the data, allowing for better comparison and processing across frames. In this study, the average values of each color channel within the ROI are normalized frame by frame, and these normalized signals are retained for subsequent analysis. This consistent ROI tracking and normalization process enables us to minimize variability and enhance the accuracy of blood pressure measurement.

### 2.2. Blind Source Separation (BSS) and Independent Component Analysis (ICA)

BSS is a technique that separates original signals from multiple observed mixed signals. It can be applied in many fields, such as image processing, telephone communications, biomedicine, speech recognition, etc. Suppose there are several different sources producing signals that are received by a receiver. The original signal can be separated from the mixed signal using these properties. This work uses ICA to achieve semi-blind source separation [12]. When the mixing matrix A is known, semi-blind source separation can calculate the unmixing matrix W more quickly, thereby separating independent component signals.

Before using independent component analysis (ICA) to separate independent components, the mixed signal undergoes essential preprocessing steps, specifically centering and whitening [13,14]. These steps simplify subsequent calculations and improve ICA’s computational efficiency by standardizing the data. Centering adjusts the data so that its mean equals zero, reducing bias and ensuring more accurate separation. This preprocessing is represented in Equation (1), where X represents the original measurement signal and $E\left[X\right]$ denotes the expected value, calculated as the weighted average of all possible values.

(1) $$\hat{X}=X-E\left[X\right]$$

Whitening transforms the data to remove correlations between variables, making them uncorrelated and with unit variance. This process enhances the accuracy of ICA in separating independent signals by ensuring that all components are equally weighted for noise reduction. Specifically, we set a threshold for noise rejection, filtering out signals with variances below this threshold. This ensures that only the significant, relevant signals contribute to the analysis, thereby improving blood pressure prediction accuracy.

By carefully applying centering and whitening, ICA effectively isolates and enhances the signals within the region of interest (ROI), enabling more reliable physiological measurement and reducing potential interference from artifact signals.

### 2.3. Nelder–Mead Particle Swarm Optimization (NM-PSO)

The principle of the NM single method is to compare each point. After each operation, each point will move toward the optimal point. First, each point is substituted into the evaluation function f, each point is ranked in order, and the best point $P_{l}$, the second-best point $P_{s}$, and the worst point $P_{h}$ are found. Then, new points are generated and compared through the four steps of reflection, expansion, contraction, and shrink. The original worst point is replaced if the better one is better. Otherwise, the following calculations are performed. The above steps are repeated until each point is very close to the optimal solution; then, the convergence condition is reached, and the calculation process ends [15,16,17].

Each population represents a possible solution to the optimization problem in cluster optimization, and each bird or fish corresponds to a particle. Each particle has the potential to become the best solution, and each particle has a corresponding objective function value, called a fitness value. The initialization phase of the PSO algorithm randomly generates a group of particles (random number). Each particle is represented by $x_{id}$, where i is the total number of particles and d represents the dimension of the particle. Then, the best solution for the group is found through an iterative process. In each iteration, the particles are updated based on the two best values: the individual best solution (Pbest) and the group best solution (Gbest). The individual best solution is the best solution by the particle itself. In contrast, the group’s best solution is the best solution found so far by the entire population. Each particle refers to Pbest and Gbest to determine the speed of the next movement based on the measurement results. The updated speed is shown in Equation (2), and the updated position is shown in Equation (3). After the new position is generated, the calculation is repeated. The optimal solution can be found after several iterations [18,19,20].

(2) $$v_{id}\left(t+1\right)=w\times v_{id}\left(t\right)+{rand}_{1}\times c_{1}\times \left(Pbest-x_{id}\right)\;+{rand}_{2}\times c_{2}\times \left(Gbest-x_{id}\right)$$

(3) $$x_{id}\left(t+1\right)=x_{id}\left(t\right)+v_{id}\left(t+1\right)$$

Among them, i is the $i_{th}$ particle; d is the $d_{th}$ dimension; t is the $t_{th}$ measurement; w is the weight value; $c_{1}$ and $c_{2}$ are the acceleration weight values; ${rand}_{1}$ and ${rand}_{2}$ are random numbers [0, 1]; $v_{id}(t)$ is the velocity of the particle at the tth measurement; $x_{id}(t)$ is the position of the particle at the tth measurement; $v_{id}(t+1)$ is the velocity of the particle at the ${t+1}_{th}$ measurement; $x_{id}(t+1)$ is the position of the particle at the ${t+1}_{th}$ measurement; Pbest is the best solution of previous measurements for each particle; and Gbest is the best solution of previous measurements for all particle groups.

**Figure 3:**
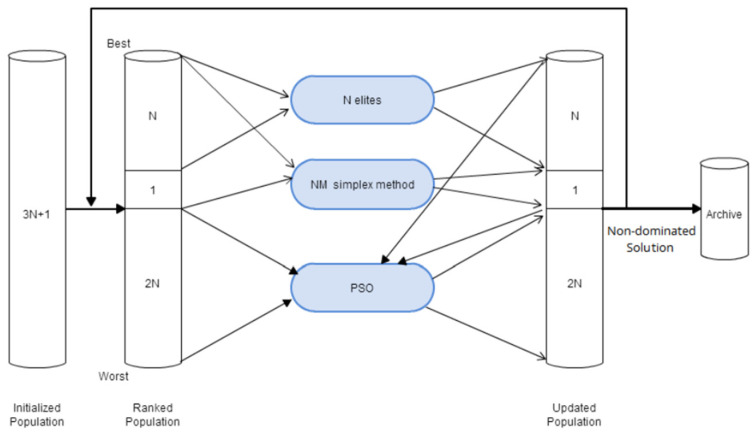
NM-PSO flow chart [21].

The concept of NM-PSO used in this work combines particle swarm optimization and Nelder–Mead monomer methods. The advantage of the NM single-body method is that it is swift during the search, but the disadvantage is that it is easy to fall into the local optimal solution. The advantage of the PSO algorithm is that it is not easy to fall into the local optimal solution, but the disadvantage is that it requires a more significant number of particle groups, resulting in the operation speed being slower. By combining the advantages of these two methods, the Nelder–Mead single-body method can be used to improve the convergence speed of PSO and reduce the number of groups. At the same time, PSO can be used to improve the dilemma that the Nelder–Mead single-body method quickly falls into the local optimal solution [21]. In the NM-PSO algorithm, to solve an N-dimensional problem, it is necessary to first generate 3N + 1 particle numbers, calculate the fitness value according to the position of each particle, and sort them, which can be divided into the first N, the first for N + 1, and the last 2N groups, and calculate the first N and N + 1 of the retained groups using the NM single-body method to obtain the updated N + 1. Then, the updated first N + 1 and the remaining 2N groups are calculated using the PSO algorithm; the first N + 1 is retained, and only the poorer 2N is updated. This process is repeated until the calculation stop condition is met, as shown in Figure 3.

### 2.4. Linear Regression

Linear regression [22,23,24,25,26] is commonly used in mathematical research methods to measure predictions and model them with multiple input variables. A data evaluation and modeling method establishes a linear relationship between dependent and independent variables. Therefore, this method can analyze and learn the relationship between the modeled dependent and independent variables in the current training results. Multivariate linear regression (MLR) is a statistical method that uses multiple independent variables to predict the outcome of a variable. MLR aims to model the linear relationship between the independent variable x and the dependent variable y to be analyzed. The basic model of MLR is Equation (4), and the matrix form is Equation (5).

(4) $$y=\beta_{0}+\beta_{1}x_{1}+\ldots +\beta_{m}x_{m}+\varepsilon$$

(5) $$y=\left[\begin{array}{c} \begin{array}{c} y\\ y_{2}\\ \vdots \end{array}\\ y_{n} \end{array}\right]=\left[\begin{array}{c} \begin{array}{c} \begin{array}{cc} \begin{array}{ccc} 1 & \begin{array}{cc} x_{11} & x_{12} \end{array} & \ldots \end{array} & x_{1i} \end{array}\\ \begin{array}{cc} \begin{array}{cc} \begin{array}{ccc} 1 & x_{21} & x_{22} \end{array} & \ldots \end{array} & x_{1i} \end{array}\\ \;\begin{array}{cc} \begin{array}{cc} \begin{array}{ccc} \vdots \;\;\; & \vdots \;\;\;\; & \vdots \end{array}\;\; & \;\vdots \;\; \end{array} & \;\;\vdots \; \end{array} \end{array}\\ \begin{array}{cc} \begin{array}{cc} \begin{array}{ccc} 1 & x_{n1} & x_{n2} \end{array} & \ldots \end{array} & x_{1i} \end{array} \end{array}\right]=\left[\begin{array}{c} \begin{array}{c} \beta_{0}\\ \beta_{1}\\ \vdots \end{array}\\ \beta_{m} \end{array}\right]+\left[\begin{array}{c} \begin{array}{c} \varepsilon_{0}\\ \varepsilon_{1}\\ \vdots \end{array}\\ \varepsilon_{m} \end{array}\right]$$

Among them, y is the strain number; $x_{1},\;x_{2},\;\ldots ,\;x_{m}$ are the  m independent variables; $\beta_{0}$ is the intercept; $\beta_{1},\;\beta_{2},\;\ldots ,\;\beta_{m}$ are the regression coefficients of the strain tree corresponding to the independent variables; and ε is the residual.

When performing multiple regression analysis, the data must meet the following characteristics: linear relationship, independence, homoscedasticity, and normality. These premise assumptions are intended to make the model more effective and can be based on the abovementioned conditions to assess whether the current data are appropriate. Next, Equation (4) is simplified to obtain Equation (6).

(6) y=xβ+ε

Next, the ordinary least squares (OLS) is used to calculate the β value, as shown in Equation (7).

(7) $$L=\sum_{i=1}^{n}({y_{i}-{\hat{y}}_{i})}^{2}$$

Among them, $y_{i}$ is the predicted result, ${\hat{y}}_{i}$ is the correct data, and a part of ${\hat{y}}_{i}$ is the so-called residual ε, so that the formula can be converted into Equation (8).

(8) $$L=\sum_{i=1}^{n}\varepsilon_{i}^{2}$$

According to the previous derivation, we know that ε is a vector, so according to the characteristics of the vector, the square of ε can be expressed as Equation (9).

(9) $$L=\sum_{i=1}^{n}\varepsilon_{i}^{2}=\varepsilon^{T}\varepsilon$$

The minimum square difference is calculated and Equation (10) is derived.

(10) $$L=\varepsilon^{T}\varepsilon =(y-x\beta {)}^{T}(y-x\beta )=y^{T}y-y^{T}x\beta -\beta^{T}x^{T}y+\beta^{T}x^{T}x\beta \;=y^{T}y-2\beta^{T}x^{T}y+\beta^{T}x^{T}x\beta$$

After taking the derivative, the results are organized as shown in Equation (11).

(11) $$\beta =(x^{T}x{)}^{-1}x^{T}y$$

Therefore, no matter how many independent variables there are today, their corresponding β values can be obtained by fitting them into the above formula. Therefore, when all beta coefficients are calculated, the original multiple regression formula can be returned and used to make predictions.

### 2.5. Principle of Non-Contact Blood Pressure Measurement

Remote photoplethysmography (RPPG) is a non-contact physiological signal measurement method using a camera. RPPG technology is based on the same optical principle as traditional PPG and detects blood volume changes in capillaries. However, unlike traditional PPG, RPPG does not require direct contact between the sensor and the skin. It extracts the pulse waveform by capturing the light reflected by the skin [27,28,29,30,31,32]. In RPPG technology, the camera captures parts such as the face or fingers and then calculates the changes in blood volume by analyzing the color changes in each image frame. Because blood flow causes tiny changes in skin color, these changes can be detected in the camera’s image. Standard RPPG technology uses green light because green light is the most sensitive to hemoglobin in the blood.

Photoplethysmography (PPG) is a non-invasive optical technique that can detect changes in blood volume in blood vessels. This technology uses the principle that the skin’s light absorption changes with blood volume pulses to measure the amount of blood flowing through the tissue with each heartbeat. When the light beam passes through the microvascular tissue, part of the light will be absorbed due to reflection or penetration by the skin tissue. The absorption rate of light can be described by Beer–Lambert Law, as shown in Equation (12) [33].

(12) $$A=-\log_{10}\frac{I_{t}}{I_{0}}=\log_{10}\frac{1}{T}=K\cdot l\cdot c$$

Among them, A is the absorbance; $I_{0}$ is the intensity of the incident light; $I_{t}$ is the intensity of the transmitted light; T is the transmittance; K is the absorption coefficient; l is the thickness of the absorbing medium; and c is the concentration of the light-absorbing substance.

In natural physiological systems, blood viscosity, thickness of the aorta, and radial vibration of the arteries in the arterial system assume that the heart is a periodic pressure pump. Therefore, Radial Resonance Theory (RRT) [34,35,36,37,38,39] was proposed, in which the arterial system is regarded as a transmission system of blood fluctuations and a combination of various inherent modes or natural vibrations. RRT can provide a model for blood pressure calculation, and by combining pulse wave signals with body mass index, an extended linear model was developed for blood pressure prediction. In RRT, the radial pulse pressure is described in Equation (13).

(13) $$p\left(z,t\right)=\sum_{k=0}^{N}\left[a_{k}\cos\left(\omega_{Hk}t\right)+b_{k}\sin\left(\omega_{Hk}t\right)\right]e^{-k\times \frac{z}{c}}$$

Among them, $\omega_{Hk}$ is the angular frequency; z is the distance between the subject and the lens; c is the wave speed; k is the number of waves; and $a_{k},\;b_{k}$ are the amplitudes of the waves.

Since the zeroth and first waveforms are the most obvious, and waveforms above the second time are omitted, Equation (13) can be simplified to Equation (14).

(14) $$p\left(t\right)=\left[a_{0}\cos\left(0\right)+b_{0}\sin\left(0\right)\right]e^{-0\times \frac{z}{c}}+\left[a_{1}cost+b_{0}sint\right]e^{-1\times \frac{z}{c}}\;=a_{0}+Q\cdot \cos\left(t\right)+R\cdot \sin\left(t\right)$$

According to Equation (14), the collected wave peak ($E_{peak}$) and wave valley ($E_{valley}$) form a pressure-related signal. With the heart’s cyclic contractile activity, the blood vessels absorb and attenuate the light. When the heart contracts, the blood vessel volume increases, the light absorption reaches the maximum, and the light reflection intensity reaches the minimum; conversely, when the heart relaxes, the blood vessel volume shrinks, the light absorption reaches the minimum, and the reflection intensity increases [40]. Peaks and troughs are closely related to blood pressure. First, the number of peaks and valleys is counted; then, the scattered peaks and valleys caused by the perturbation are removed. Assuming a sampling frequency of 20 FPS and a heart rate of 120 beats per minute, a heartbeat every 0.5 s, 0.5 × 20 equals 10 data points. The interval between peaks and troughs is 0.25 s. The data are discarded if the interval between any pair of peaks and troughs is less than 0.25 s. Once the peak and trough values of the signal are obtained, their average value is calculated, as shown in Equations (15) and (16).

(15) $$E_{peak}=\frac{\Sigma_{n_{1}}H_{D}}{n_{1}}$$

(16) $$E_{valley}=\frac{\Sigma_{n_{2}}H_{L}}{n_{2}}$$

Among them, $n_{1}$ and $n_{2}$ are the number of peaks and troughs; $H_{D}$ is the sequence value of the peak; and $H_{L}$ is the sequence value of the valley.

## 3. Research Methods and System Description

Both methods in this work analyze blood pressure by capturing the rPPG signal reflected by light from the palm. This chapter discusses the system architecture, introduces the research and measurement environments, and describes the algorithm flow used in this work. In this study, we selected the NM-PSO and regression methods due to their complementary strengths in non-contact blood pressure measurement. The NM-PSO method was chosen for its robustness in noise handling and its ability to optimize complex parameters in real-time, which is essential in non-contact settings where signal quality may vary. The regression method, on the other hand, was selected for its computational efficiency, allowing for rapid blood pressure estimation through a predefined formula. By comparing these two methods, we aim to evaluate their respective advantages in terms of accuracy, processing speed, and robustness to noise, providing insights into their applicability in real-world scenarios.

### 3.1. System Architecture

**Figure 4:**
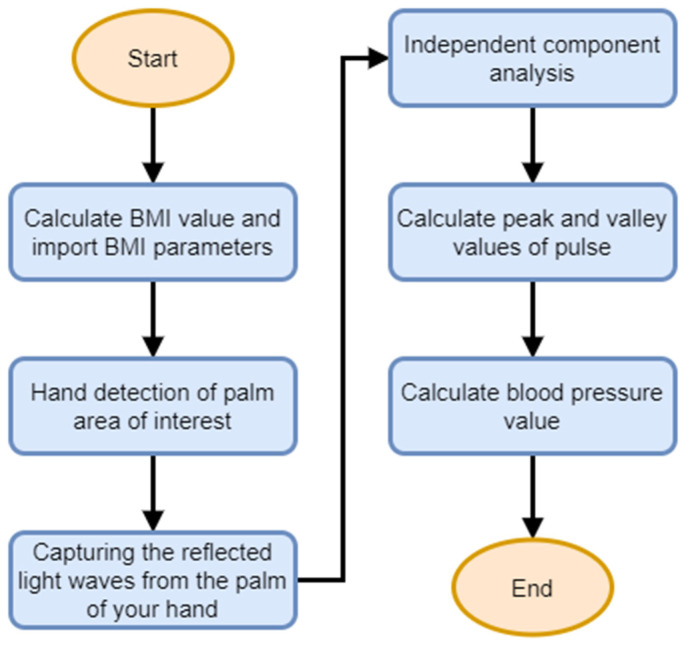
NM-PSO method blood pressure measurement system flow chart.

This work proposes two non-contact blood pressure measurement systems to help people monitor their blood pressure values at any time. There is no need to use bulky and expensive blood pressure instruments, as they can only be measured through a general webcam. First, the NM-PSO method is introduced. Figure 4 shows the flow chart of the NM-PSO method’s non-contact blood pressure measurement system. The system architecture is as follows: The tester enters the height and weight on the computer to calculate the BMI value and finds the corresponding value in the table. The blood pressure formula parameters of the BMI range are then used for hand detection, capturing the light waves reflected on the palm through the area of interest and substituting the extracted values and empirical parameters into the formula to calculate the blood pressure value.

**Figure 5:**
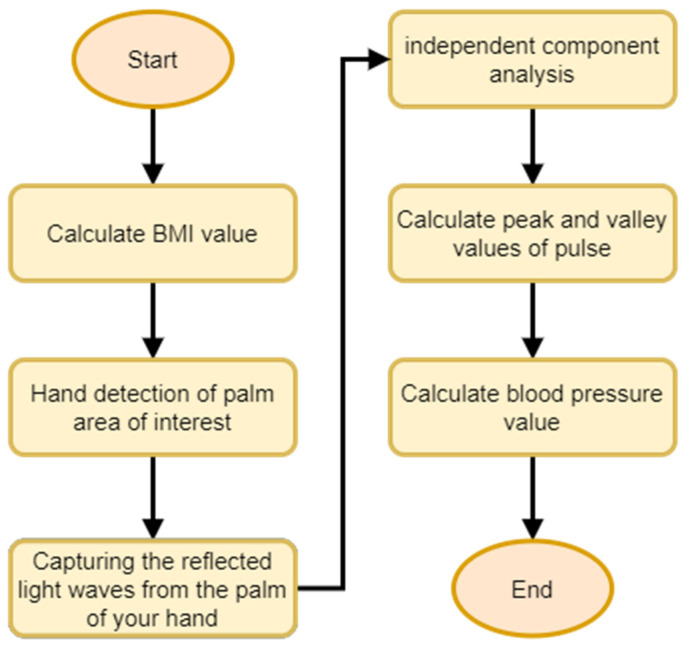
Regression method blood pressure measurement system flow chart.

The second method is the regression method. Figure 5 shows the flow chart of the regression method’s non-contact blood pressure measurement system. The system architecture is as follows: First, the tester enters the height and weight on the computer to calculate the BMI value and then uses a webcam to perform hand detection. To measure, the light waves reflected on the palm through the area of interest are obtained, the peak and trough values are removed and combined with the BMI value, and then substituted into the formula calculated through machine learning to calculate the blood pressure value.

### 3.2. Experimental Environment

This aim of this research is to capture images through a webcam while the computer performs signal processing based on the captured images. The light source is a fluorescent lamp with 500 to 650 lumens, and the experimental distance is about 15 cm. In order to verify the accuracy of the experimental data, a contact blood pressure machine was used for comparison during measurement. Table 2 shows the environment construction and hardware architecture of this work.

### 3.3. Blood Pressure Formula Empirical Parameters

This work established a table of empirical parameters of the blood pressure formula with BMI values as intervals. The empirical parameters of the blood pressure formula in this system can be divided into the following steps:

1. The test taker first uses a blood pressure machine to measure blood pressure. Then, the test taker enters the height and weight on the computer to calculate the BMI value. Then, the webcam captures the area of interest on the hand and extracts the pulse wave signal from the palm part.
2. The signal is normalized, and independent component analysis is used to remove waveform artifacts.
3. The NM-PSO algorithm is used to calculate the empirical parameters in the formula.
4. The empirical parameters are classified into BMI, intervals and the empirical parameters in each interval are tested as the formula parameters for that interval.

#### 3.3.1. Blood Pressure Formula Parameter Calculation for NM-PSO

This work uses the blood pressure formula proposed in the references [41] to calculate blood pressure values, as shown in Equation (17).

(17) $$f\left(x,BMI\right)=a_{0}+a_{1}\cdot x+a_{2}\cdot BMI+a_{3}\cdot x\cdot BMI$$

In order to speed up the calculation of blood pressure, this work improved Equation (17) and derived a new formula, as shown in Equation (18)

(18) $$f\left(x,BMI\right)=\alpha_{0}+\alpha_{1}\cdot x+BMI\times \left(1+\alpha_{2}\cdot x\right)$$

Among them, BMI is the body mass index of the tester; x is the average of all peaks or valley; and $\alpha_{0}\sim \alpha_{2}$ are empirical parameters.

Before calculating the empirical parameters, the BMI value of the test subject was calculated, and a blood pressure machine was used to measure the blood pressure. At the same time, the peak ($E_{peak}$) and valley ($E_{valley}$) of the pulse wave signal on the palm were extracted through the webcam on the computer. Then, the experiment used the person’s BMI value, the value measured by the blood pressure machine, and the extracted $E_{peak}$ and $E_{valley}$, which were substituted into the adaptation function Equations (19) and (20), and the NM-PSO algorithm mentioned in Section 2.3 of this paper was used to calculate the output of the empirical parameters $\alpha_{0}\sim \alpha_{2}$. The relevant setting parameters of the NM-PSO algorithm are shown in Table 3.

(19) $$SBP=\alpha_{0}+\alpha_{1}\times E_{peak}+BMI\times \left(1+\alpha_{2}\times E_{peak}\right)$$

(20) $$DBP=\alpha_{0}+\alpha_{1}\times E_{valley}+BMI\times \left(1+\alpha_{2}\times E_{valley}\right)$$

Among them, BMI is the body mass value of the tester; $E_{peak}$ and $E_{valley}$ are the average values of all peaks or valley; and $\alpha_{0}\sim \alpha_{2}$ are empirical parameters.

#### 3.3.2. Blood Pressure Formula Parameter Calculation for Linear Regression

In the regression method, the BMI value of the test subject was first calculated, and the blood pressure was measured using a blood pressure machine. At the same time, the peak ($E_{peak}$) and valley ($E_{valley}$) of the pulse wave signal on the palm were extracted through the webcam on the computer. Then, the experimenter used the BMI value, measured by the blood pressure machine, and the extracted $E_{peak}$ and $E_{valley}$ were calculated using regression calculation to calculate the parameters $\gamma_{0}\sim \gamma_{2}$, as shown in Equations (21) and (22).

(21) $$SBP=\gamma_{0}+\gamma_{1}\times BMI+\gamma_{2}\times E_{peak}$$

(22) $$DBP=\gamma_{0}+\gamma_{1}\times BMI+\gamma_{2}\times E_{valley}$$

Among them, BMI is the body mass value of the tester; $E_{peak}$ and $E_{valley}$ are the average values of all peaks or valley; and $\gamma_{0}\sim \gamma_{2}$ are empirical parameters.

### 3.4. Experimental Steps

**Figure 6:**
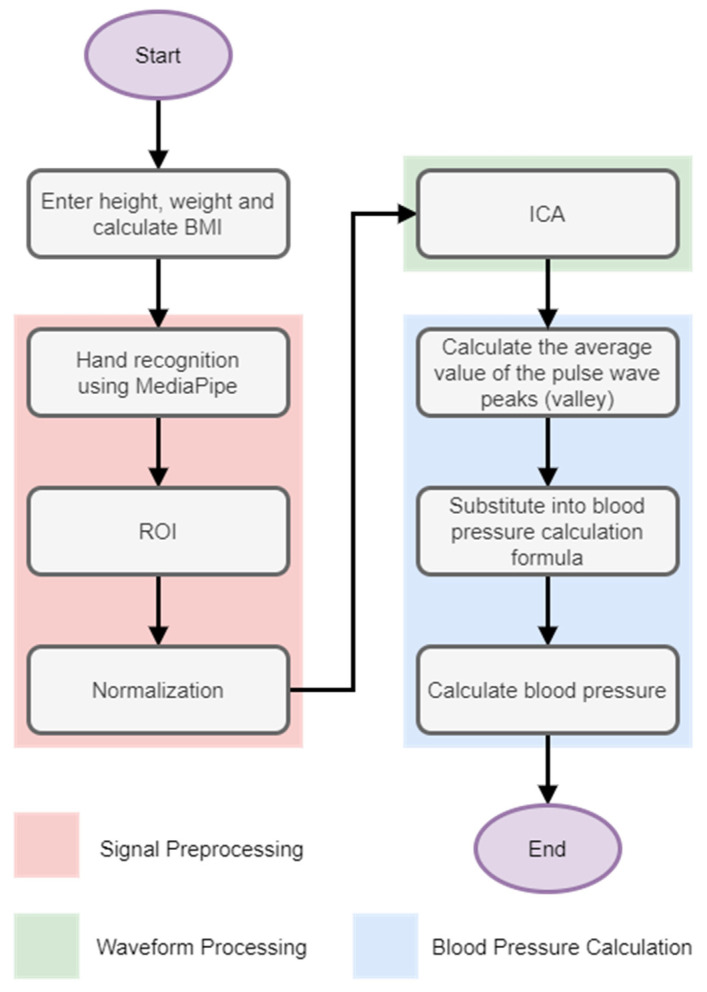
Non-contact blood pressure measurement flow chart.

The non-contact blood pressure measurement process of the NM-PSO and regression methods is mainly divided into the following four steps. The flow chart is shown in Figure 6.

**Figure 7:**
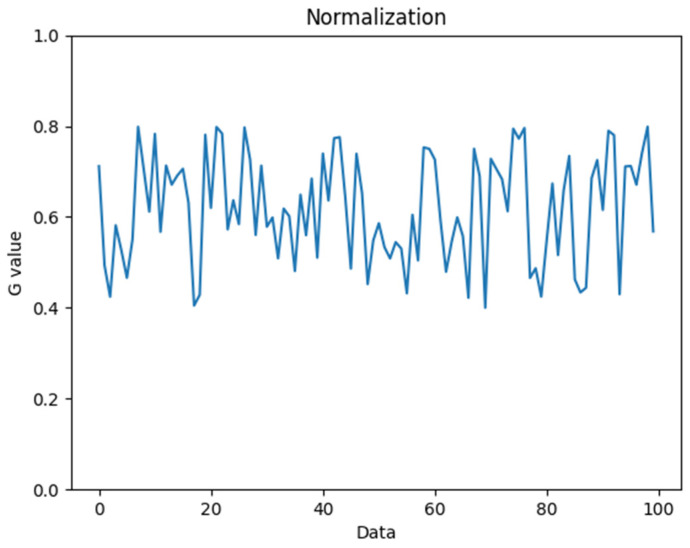
Normalized waveform analysis chart.

1. First, the tester’s height and weight were used to calculate the BMI value to be substituted into the blood pressure formula.
2. The tester captured the hand image through a webcam and used MediaPipe hand recognition to intercept the pulse wave signal of the palm of his hand through the area of interest.
3. The signal was normalized to adjust itself, and then independent component analysis was used to remove waveform artifacts.
4. Finally, the peaks and troughs of the pulse wave and the BMI value after removing the waveform artifacts into the blood pressure formula were substituted to calculate the blood pressure.

- Signal Preprocessing
  This work used the MediaPipe hand 21-point features to identify the hand in the image and capture the palm part through the area of interest. In order to improve the performance and speed of subsequent calculations, regularization was performed, as shown in Figure 7.
- Waveform Processing
  This work used independent component analysis to process the extracted pulse waves to eliminate noise interference caused by ambient lighting and human body shaking often encountered in RPPG measurements. The following are detailed process steps for independent component analysis:
  1. Measurement signal X is inputted.
  2. Centralization: the input signal is averaged, $\hat{X}=X-E\left[X\right]$.
  3. Whitening: $Z=V\hat{X}=VAS=\tilde{A}S$.
  4. The number n of independent components is selected for estimation.
  5. Initial $W_{i},\;i=1,\ldots ,n$ is randomly selected.
  6. At the same time, Newton’s method is iteratively updated for each $W_{i}$: Objective function: $Max\{E\left[G\left({W_{i}}^{T}Z\right)\right]-E\left[G\prime \left({W_{i}}^{T}Z\right)\right]W_{i}\}$
  7. Convergence criterion: Whether the maximum value of iterations has been reached based on the number of iterations set by the user is checked. If the maximum value is reached, output W and end. If the convergence condition is not reached, step 5 is repeated, and iteration is continued until the condition is met.
  8. The independent component signal Y is found.
- Blood Pressure Calculation
  A total of 10 peak (valley) values was taken in a blood pressure measurement. Finally, the average of these ten values was used as the measurement’s peak (valley) value for blood pressure calculation. The following are the steps to obtain the first peak (valley) value:
  1. First, the system extracts all the values of the peaks (valleys) of the pulse wave within 1 s.
  2. According to Equations (15) and (16) in this article, after adding up all the peak (valley) values and removing the number of values, the peak (valley) value of the first piece of data can be obtained.
  According to the above steps, this study continuously obtained ten pieces of data and then averaged the peak (valley) values obtained from each piece of data to calculate the peak (valley) value required for a blood pressure measurement.
  Calculating blood pressure values using the NM-PSO method: The empirical parameters obtained by NM-PSO, the average peak (valley), and the BMI value were substituted into this article’s Equation (18) for calculation.
  Regression method to calculate blood pressure value: BMI, empirical parameters, and peak (valley) values were used and substituted into Equations (21) and (22) in this article for linear regression calculation.

## 4. Results

The study measured 55 volunteer participants. While performing non-contact blood pressure measurements, the tester used a blood pressure machine certified by Taiwan’s Ministry of Health and Welfare to measure as a standard and compared the two values for error verification. This research captured the RPPG signal reflected by light from the palm. To reduce interference from other factors, the measurement environment was set to a distance of about 15 cm and an illumination of 550 lux. Using the mean absolute error (MAE), the mean absolute percentage error (MAPE), and the root mean square error (RMSE) as the evaluation indicators of accuracy, the Equations are as follows: (23), (24), and (25).

(23) $$MAE=\frac{1}{N}\sum_{i=1}^{N}\left|A_{i}-F_{i}\right|$$

(24) $$MAPE=\frac{1}{N}\sum_{i=1}^{N}\frac{\left|A_{i}-F_{i}\right|}{A_{i}}\times 100\%$$

(25) $$RMSE=\sqrt{\frac{1}{N}\sum_{i=1}^{N}{\left(F_{i}-A_{i}\right)}^{2}}$$

Among them, N is the total number of data points; $A_{i}$ is the i-th observed value (actual value); and $F_{i}$ is the i-th predicted value.

**Figure 8:**
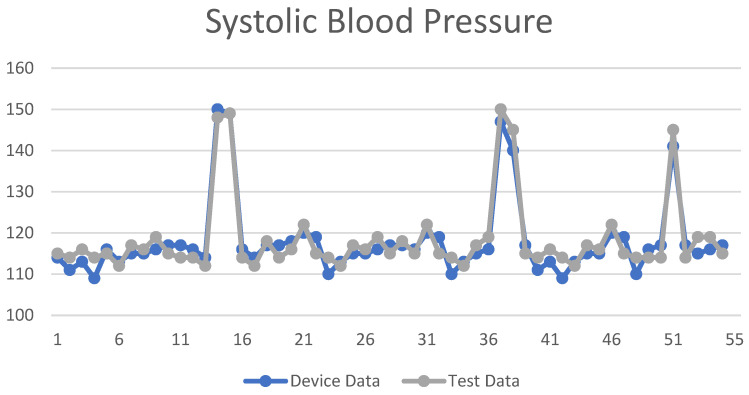
SBP data line chart using the NM-PSO method.

**Figure 9:**
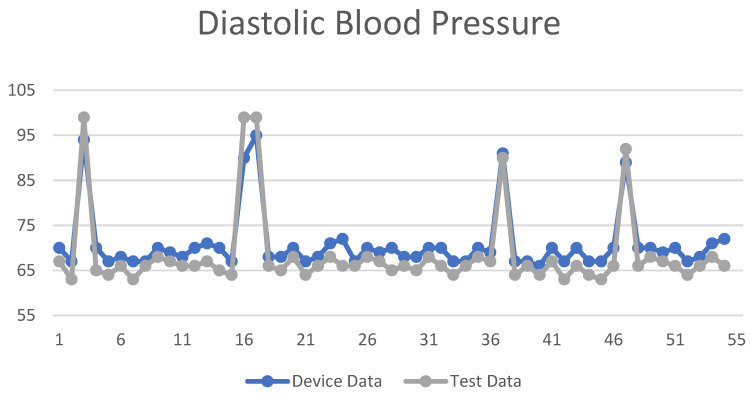
DBP data line chart using the NM-PSO method.

The NM-PSO method was used to compare the measurement values of the blood pressure measuring instrument. The SBP and DBP measurement values are shown in Figure 8 and Figure 9. The *X*-axis is the number of test subjects, and the *Y*-axis is the blood pressure value.

**Figure 10:**
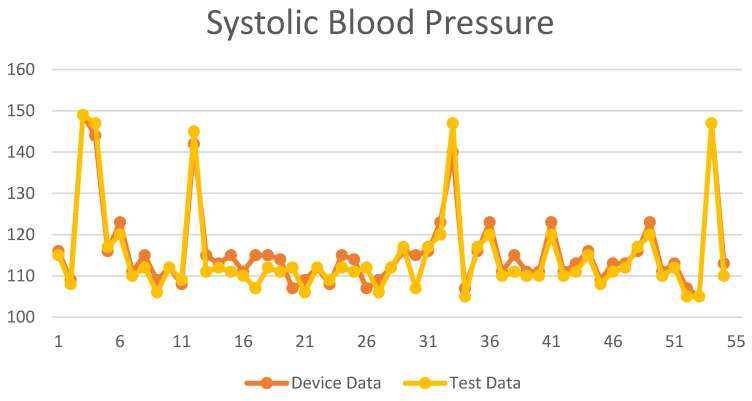
SBP data line chart using the regression method.

**Figure 11:**
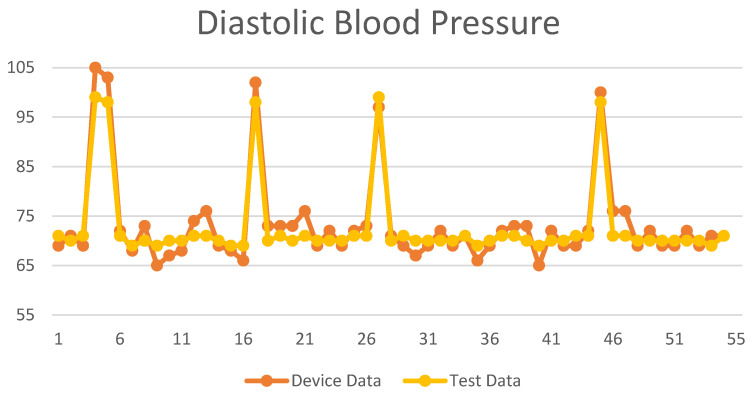
DBP data line chart using the regression method.

The regression method was used to compare the measurement values of the blood pressure measuring instrument. The SBP and DBP measurement values are shown in Figure 10 and Figure 11. The *X*-axis is the number of test subjects, and the *Y*-axis is the blood pressure value.

In this work, the accuracy of the NM-PSO method was that the MAE of SBP was 2.44 mmHg, the MAPE was 2.06%, and the RMSE was 2.71 mmHg; the MAE of DBP was 3.09 mmHg, the MAPE was 4.51%, and the RMSE was 3.42 mmHg. As well as the accuracy of the regression method, the MAE of SBP was 2.22 mmHg, MAPE was 1.94%, and RMSE was 2.88 mmHg; the MAE of DBP was 2.20 mmHg, MAPE was 2.99%, and RMSE was 2.60 mmHg, as shown in Table 4.

Table 5 presents the 95% confidence intervals for systolic blood pressure (SBP) and diastolic blood pressure (DBP) measurements obtained from both the NM-PSO and regression methods. For the device data, the mean SBP is 117.93 mmHg with a 95% confidence interval of (115.43, 120.43) mmHg, and the mean DBP is 71.5 mmHg with a confidence interval of (69.5, 73.5) mmHg. In the test data, the mean SBP is 118.47 mmHg with a confidence interval of (115.89, 121.06) mmHg, while the mean DBP is 69.8 mmHg with a confidence interval of (67.9, 71.8) mmHg. These confidence intervals indicate the range within which the true values of blood pressure are expected to fall for both methods, underscoring the reliability and consistency of the measurements achieved by the two approaches.

## 5. Discussion

In evaluating the performance of the blood pressure method presented in this paper, we considered widely accepted standards for the approval of blood pressure measurement devices used in clinical settings. The Association for the Advancement of Medical Instrumentation (AAMI) standards are developed to assist medical device companies. Products manufactured meet global standards for the safe use of medical devices.

The AAMI standard stipulates that compared with the gold standard measurement, the average absolute error of the device shall not exceed 5 mmHg, and the standard deviation (SD) shall not exceed 8 mmHg. If the device meets the above standards, it is rated as a “PASS”; otherwise, it is rated as a “FAIL”. Table 6 shows the comparison with AAMI standards.

Moreover, to ensure this work’s accuracy and compare it with related references, Amal E. et al. [1] proposed a multi-stage model based on a deep neural network, using a PPG signal to estimate systolic and diastolic blood pressure. Nicolas A. et al. [2] proposed a cuffless method to monitor arterial blood pressure using a deep learning model based on seq2seq architecture with an attention mechanism to estimate the shape of the average pulse. This method requires only the raw PPG signal from the finger. Aguirre N. et al. [3] is a camera-based image sequence that can achieve contactless and continuous blood pressure measurement through forehead imaging iPPG and proposes a new deep learning method to achieve blood pressure detection. Stogiannopoulos et al. [4] used infrared cameras and motion magnification techniques to enhance subtle changes in skin pixel intensity for estimating blood pressure. Fang et al. [5] employed a deep learning model with CNN+BiLSTM+GRU to improve the accuracy of IPPG signal processing and used multi-layer filtering techniques to reduce noise. Cheng et al. [6] proposed a multi-stage model that combined CNN and Bi-GRU for blood pressure estimation; the comparison results of this work and references [1,2,3,4,5,6] are shown in Table 7.

Some studies used PPG-based methods, which require direct contact sensors to capture blood pressure signals, providing accurate results but often lacking practicality for non-contact applications. Other studies utilized iPPG, which achieves high accuracy by capturing signals from facial or forehead regions. However, iPPG methods generally need controlled lighting conditions and specialized equipment, making them less suitable for everyday use. In contrast, our study employed RPPG to capture signals from the palm using a standard webcam, providing a non-contact, cost-effective solution that also reduces measurement time. This approach allows for greater accessibility and flexibility, enhancing its suitability for daily health monitoring applications, especially in home environments.

In addition to comparing our methods with other studies, we acknowledge certain limitations inherent in non-contact blood pressure measurement. Environmental factors, such as changes in lighting conditions and hand movement, can affect signal stability and measurement accuracy. The NM-PSO method, while robust in handling noise, may require more computational resources compared to the regression method, which provides faster estimations but may be more susceptible to environmental fluctuations. Each method has specific strengths—NM-PSO offers higher noise resilience, while the regression method excels in computational efficiency—making them suitable for different application contexts. Future work could explore further improvements in adaptability to various environmental conditions.

## 6. Conclusions

This work introduces the innovative application of NM-PSO and regression methods for blood pressure calculation, achieving a significant improvement in convergence speed while effectively balancing the trade-off between speed and accuracy, which has been a limitation in existing algorithms. Compared to other approaches, our method offers notable advantages in accuracy, reduced measurement time, and lower equipment costs. By selecting the palm as the area of interest, we accelerate the acquisition of RPPG signals, enabling efficient detection of high blood pressure values exceeding 140 mmHg. This breakthrough enhances the practicality of non-invasive blood pressure monitoring and represents a substantial advancement in biomimetic engineering.

Future work could involve testing with larger and more diverse populations and evaluating the method’s robustness across various environmental conditions. Additionally, the potential for integration with consumer devices, such as smartphones, holds promise for expanding accessibility to non-invasive blood pressure monitoring, making this method an appealing option for daily health monitoring. Furthermore, we plan to extend our research to explore non-invasive methods for measuring other important health indicators, including blood glucose and cholesterol levels, to provide a more comprehensive tool for personal health management.

## Figures and Tables

**Table 1:** MediaPipe hand feature point table.

Number	Feature Point Name
0	WRIST
1	THUMB_CMC
2	THUMB_MCP
3	THUMB_IP
4	THUMB_TIP
5	INDEX_FINGER_MCP
6	INDEX_FINGER_PIP
7	INDEX_FINGER_DIP
8	INDEX_FINGER_TIP
9	MIDDLE_FINGER_MCP
10	MIDDLE_FINGER_PIP
11	MIDDLE_FINGER_DIP
12	MIDDLE_FINGER_TIP
13	RING_FINGER_MCP
14	RING_FINGER_PIP
15	RING_FINGER_DIP
16	RING_FINGER_TIP
17	PINKY_MCP
18	PINKY_PIP
19	PINKY_DIP
20	PINKY_TIP

**Table 2:** Environment setup and hardware architecture table.

Parameters	Values
Operating system	Windows 10 Professional (x64)
CPU	Intel(R) Core (TM) i7-9700 CPU @ 3.00 GHz 3.00 GHz
RAM	16 GB
Development Environment	Tensorflow-Keras (Spyder4.2.0)
Programming Language	Python3.8.8
Blood Pressure Machine	FDK FT-C12B
Webcam	Live Streamer CAM 313
Light Meter	Konica Minolta T-10

**Table 3:** NM-PSO algorithm-related parameter setting table.

Parameters	Values
Reflection Parameter (α)	1
Expansion Parameter (γ)	2
Shrinkage Parameter (β)	0.5
Acceleration Factor ($c_{1}$, $c_{2}$)	1.5
Weight (w)	0.5
Number of Particles	10
Number of Iterations	100
Maximum and Minimum Speed	120/0
Maximum and Minimum Boundary	120/0
Random Numbers (${rand}_{1}$, ${rand}_{2}$)	(0, 1)

**Table 4:** Accuracy rate table of this work.

	NM-PSO Method		Regression Method	
	SBP	DBP	SBP	DBP
MAE (mmHg)	2.44	3.09	2.22	2.20
MAPE (%)	2.06	4.51	1.94	2.99
RMSE (mmHg)	2.71	3.42	2.88	2.60
Measurement Time	10 s			

**Table 5:** The confidence intervals for SBP and DBP.

	Device Data		Test Data	
	SBP	DBP	SBP	DBP
Mean	117.93	71.5	118.47	69.8
95% Confidence Interval	(115.43, 120.43)	(69.5, 73.5)	(115.89, 121.06)	(67.9, 71.8)

**Table 6:** Comparison table with AAMI standards.

	NM-PSO Method		Regression Method	
	SBP	DBP	SBP	DBP
MAE (mmHg) ≤5	2.44	3.09	2.22	2.20
SD (mmHg) ≤8	2.65	2.51	2.65	2.54
Rating	PASS	PASS	PASS	PASS

**Table 7:** Related reference comparison table.

Model	Reference	SBP			DBP		
		MAE (mmHg)	MAPE (%)	RMSE (mmHg)	MAE (mmHg)	MAPE (%)	RMSE (mmHg)
CNN-LSTM	[1]	4.25		5.91	2.18		2.96
GRU	[2]	12.08		15.67	5.56		7.32
D1DC-LSTM	[3]	10.10		9.42	8.67		6.19
Hybrid D1DCnet	[3]	8.36		6.22	5.69		3.97
Infrared Imaging	[4]	12.01		8.43	6.38		3.91
NN+BiLSTM+GRU	[5]	12.40		17.12	5.74		8.36
Single CNN	[6]	8.24			6.42		
Dual-Layer BiGRU	[6]	7.57			5.76		
Multi-Stage Model	[6]	5.33			3.92		
NM-PSO Method	[This work]	2.44	2.06	2.71	3.09	4.51	3.42
Regression Method	[This work]	2.22	1.94	2.88	2.20	2.99	2.60

## Data Availability

The original contributions presented in the study are included in the article, further inquiries can be directed to the corresponding author.
